# Polyploidy in chronic lymphocytic leukemia with p53 deletion detected by fish: a case report

**DOI:** 10.4076/1757-1626-2-8872

**Published:** 2009-09-11

**Authors:** Maha Eid, Hesham Kayed, Hala T El-Bassyouni

**Affiliations:** 1 Department of Cytogenetics, National Research Centre, El-Tahreer Street, Dokki, Cairo, Egypt; 2 Department of Clinical Genetics, Division of Human Genetics and Genome Research, National Research Centre, El-Tahreer Street, Dokki, Cairo, Egypt

## Abstract

We report a case of chronic lymphocytic leukemia with a characteristic cytogenetics finding detected by fluorescent in situ hybridization. This case has deletion in p53 gene in 50% of interphase nuclei studied in the peripheral blood and polyploidy in 30% of cells. To our knowledge polyploidy is not commonly reported with chronic lymphocytic leukemia patients.

## Introduction

Chronic lymphocytic leukemia (CLL) is a hematopoietic neoplasm of B-lymphocytes found in the peripheral blood, bone marrow, and/or lymph nodes [1]. CLL is the most common leukemia of adults [2]. Cytogenetic study is useful in predicting clinical outcome. Abnormalities of chromosomes 11 and 17 are associated with poor prognosis; deletion of 13q is said to have a good prognosis. Patients are usually asymptomatic at presentation. Many cases present incidentally with peripheral blood lymphocytosis. Approximately 80% of patients have associated lymphadenopathy; approximately 50% will have an enlarged liver and/or spleen [3].

Loss of tumor protein 53 (TP53) has been associated withaggressive disease and poor response to therapy in B-cell chronic lymphocytic leukemia (B-CLL). TP53 is located at chromosome band 17p13 and its absence can be detected by fluorescence in situ hybridization (FISH) in the interphase nuclei of 8-10% patients with B-CLL [4,5]. Previous studies showed that p53 plays a central role in G1 and DNA damage checkpoints, thus contributing to genomic stability [6].

## Case presentation

A 60-year Egyptian female presented with splenomegaly and lymphadenopathy. The laboratory tests showed Hb: 11 g/dL, TLC: 12 × 10^9^/L, PLT: 130 × 10^9^/L, absolute lymphocyte: 8 × 10^9^/L, relative lymphocyte: 73 × 10^9^/L, bone marrow lymphocyte: 78 × 10^6^/mL. Biochemical test revealed slight increase in the lactic dehydrogenase (LDH): 260 IU/l (normal up to 240 IU/L) and Ca: 7.5 mg/dL. The lymphocytes of the bone marrow express an immunophenotype suggestive of B-cell lymphoproliferative type. The immunophenotype of leukemic cells was CD19: 68%, CD22: 60%, CD22: 57%, CD23: 52%, CD79b: 58.5%. The myeloid markers were negative. Also HLA-DR was 71% and CD38 was 58.2%.

The cytogenetic analysis of the peripheral blood revealed unsuccessful culture for chromosomal study. FISH study was done using (DLEU (13q14)/p53 (17p13) red/green Kreatech) probe, the FISH revealed deletion in p53 in 50% of cells, and 3-5 copies of 13q14 in 30% of cells. First, it was thought that there is gene amplification in this region, which is a very rare finding. So, FISH study was done using (Sub Telomere 13qter green) probe to exclude polosomy. This also revealed 3-5 signals in 30% of cells. Then polyploidy was suggested and confirmed by (Sub Telomere 1pter green) which also gave 3-5 copies of 13q14 in 30% of cells.

## Discussion

Polyploidy could be explained by the deletion in the p53 gene which is essential in the regulation of cell cycle and mitotic division. There is much evidence linking p53 and development of polyploidy [7]. Recently, it was shown that the transcriptional induction of p53 by mitotic checkpoint activation is essential in protecting cells from developing abnormal levels of chromosome ploidy caused by mitotic failure. Studies have shown that p53 deficiencies induce insufficient mitosis arrest, compromise apoptosis, and can cause profound aneuploidy. Despite this, the molecular mechanisms implicating p53 mitotic regulation with a chromosomal instability phenotype is not yet clearly demonstrated. However, this might be accompanied by other gene deletion such as p73 that is also key regulatory genes in between chromosomal instability and cancer development. It should be realized that p53 defects could lead to highly unstable karyotypes that might ultimately push cells towards malignant transformation (6). Moreover, CLL patients with *P53* gene deletion progress rapidly, respond poorly to therapy, and do not survive for long [8,9].

## Conclusion

In conclusion, the genetic instability is caused by deletion in the p53 gene, the factor that led to excessive DNA replication. To our knowledge polyploidy is not commonly reported with CLL patients.

## Patient's perspective

I suffered from leukemia, and then I was referred to do cytogenetic analysis which revealed a chromosomal defect. Then I was given genetic counseling and was told that I need follow up.

**Figure 1:**
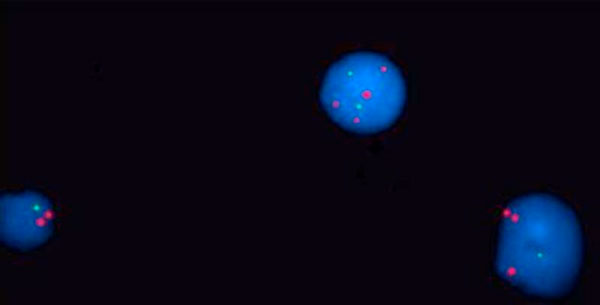
**Deletion in p53 (green) and multiple copies of 13q14 (red)**.

**Figure 2:**
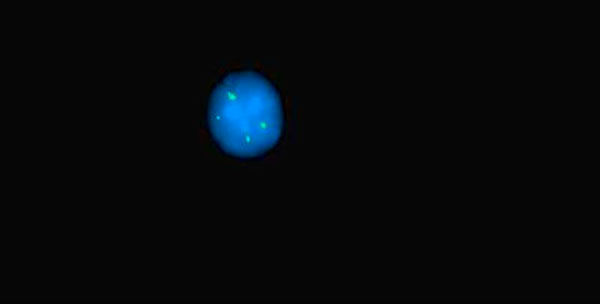
**4 signals of sub telomere 1p**.

**Figure 3:**
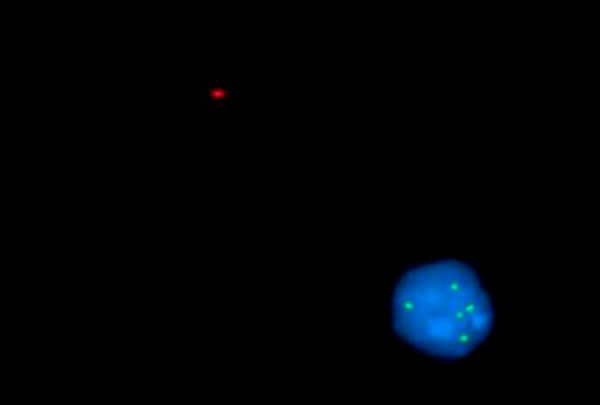
**Five copies of sub telomere 13q**.

## Abbreviations

B-CLL: B-cell chronic lymphocytic leukemia; CLL: chronic lymphocytic leukemia; FISH: fluorescence insitu hybridization; Hb: hemoglobin; LDH: lactic dehydrogenase; PLT: platelets.

## Consent

Written informed consent was obtained from the patient for publication of this case report. A copy of the written consent is available for review by the Editor-in-Chief of this journal.

## Competing interests

The authors declare that they have no competing interests.

## Authors' contributions

ME diagnosed and followed up the patient and involved in writing the manuscript. HFK diagnosed and followed up the patient and involved in revising the manuscript. HTE contributed to conception and design of the study, involved in drafting the manuscript, and revised it. Authors read and approved the final manuscript.
